# Identifying barriers to physical activity participation and engagement among college students in Riyadh (Saudi Arabia): gender differences in perceived barriers

**DOI:** 10.3389/fspor.2025.1657854

**Published:** 2025-09-11

**Authors:** Pablo Prieto-González, Claire Alkouatli

**Affiliations:** ^1^ Sport Sciences and Diagnostics Research Group, College of Humanities and Sciences, Prince Sultan University, Riyadh, Saudi Arabia; ^2^ Educational Research Lab, College of Humanities and Sciences, Prince Sultan University, Riyadh, Saudi Arabia

**Keywords:** physical activity, gender differences, college students, Saudi Arabia, social and cultural factors, environmental barriers, health-related barriers, social support

## Abstract

**Objective:**

Despite growing public health efforts worldwide, many young adults—particularly women—remain insufficiently active, often due to a complex interplay of personal, social, and environmental barriers. In the context of rapid sociocultural change in Saudi Arabia, this descriptive study aims to identify and compare perceived barriers to physical activity (PA) among male and female college students in Riyadh across key domains.

**Methods:**

This cross-sectional observational study included 437 college students (219 females and 218 males), aged 18–25 and residing in Riyadh. Data were collected in person using a validated, self-administered questionnaire comprising 39 items across nine domains assessing barriers and behavioral aspects related to PA.

**Results:**

Significant gender differences emerged in barriers such as harassment, lack of friend support, body image concerns, safety, weather, and the absence of same-gender role models. No differences were observed in PA frequency. Key factors negatively affecting PA frequency included competing priorities, weather, transportation, and fatigue. Engagement in more structured or competitive activities was hindered by environmental pollution, time management challenges, limited group support, lack of skills, and absence of role models. However, effect sizes were generally small, and the predictive power of the models was modest.

**Conclusion:**

Gender disparities in perceived barriers were most pronounced in personal, sociocultural, environmental, and health-related domains, influencing the type of PA more than its frequency. Although some gender differences were statistically significant, their effect sizes are small and should be interpreted with caution. Promoting equitable participation requires safe, female-friendly environments, stronger peer and role model support, and improved access to equipment and green spaces. Addressing body image, personal safety, confidence, skills, and time management is also essential. Given the modest predictive power of the models, other factors likely influence PA behaviors and warrant further investigation.

## 1 Introduction

Physical activity (PA) is essential for promoting health and improving quality of life, offering numerous physical and mental health benefits (1). The World Health Organization recommends that adults practice at least 150 min of moderate-intensity PA weekly (2). However, despite these guidelines, a large portion of the population remains inactive, highlighting the urgent need to identify barriers that prevent individuals from participating in physical exercise. Identifying these obstacles is necessary when designing interventions to enhance participation in physical activities (3).

Research indicates that women face unique challenges that significantly hinder their participation in PA. Previous studies have shown that cultural traditions, societal expectations, and gender norms may influence women's experiences of PA. Huang et al. demonstrated how gender norms and stereotypes influence the perception of exercise, making it difficult for women to prioritize PA (4). Many women struggle to find time to exercise due to work and family responsibilities, limiting their ability to lead an active lifestyle. As a result, women often feel pressure to conform to societal standards of appearance and body image, resulting in less motivation to engage in PA than men (5).

In addition to cultural expectations, logistical barriers also significantly affect women's participation in PA. Previous studies revealed that many women lack access to gender-specific facilities, face transportation difficulties, and receive limited social support. Research by Asghar et al. in Pakistan revealed that more than half of young women did not participate in organized physical activities due to these factors (6). Similarly, Whipple et al. identified lack of time and social support as the most common barriers to women's PA, reflecting the need for a comprehensive approach to addressing these issues (7).

Given these complexities, understanding the barriers to PA in Saudi Arabia is particularly relevant. The interaction between traditional Arab–Muslim cultural values and the country's Vision 2030 initiative—which promotes health and well-being—creates a unique sociocultural context for examining women's participation in PA. While traditional norms may limit women's involvement in certain aspects of public life, including sports, the ongoing societal transformation driven by modernization offers an opportunity to explore how these evolving dynamics influence women's engagement in PA (8, 9).

Focusing on college-aged individuals is particularly relevant in this context, as emerging adulthood is characterized by significant lifestyle changes and identity exploration (10). This is a critical period in which long-term healthy habits are formed. However, participation in physical activity tends to decline due to increased academic demands, greater responsibilities for daily tasks such as meal preparation and time management, and new social pressures (11). These changes can directly affect PA levels. Therefore, studying this group offers valuable insight into how these transition factors influence behavior and can help design targeted, age-appropriate interventions (12).

To effectively understand the cultural issues surrounding PA in Saudi Arabia, it is essential to use a questionnaire tailored to the country's sociocultural context. This approach would identify the specific barriers women face in participating in PA. Identifying these barriers will contribute to a deeper understanding of gender disparities in sports participation and provide policymakers and community leaders with valuable information. Previous studies have utilized questionnaires to evaluate various dimensions that influence female participation, including social, personal, cultural, familial, and economic factors. Farzaneh et al. (13) used a survey developed by Mirghafouri et al. (14) to identify barriers to female participation in sports activities. The instrument consists of 30 questions, divided into five dimensions: social, personal, cultural, familial, and economic, measured using a five-point Likert scale. Similarly, Sam et al. used the “Barriers to Physical Activity and Exercise Participation” questionnaire, identifying the main external and internal barriers among respondents (15). Justine et al. (16) used a 45-item questionnaire, while Kanwar and Kaur (17) and van Wyk (18) relied on direct interviews to obtain qualitative information.

However, despite the growing body of literature exploring barriers to PA, significant gaps remain in studies focusing on young adults in rapidly changing sociocultural environments, such as Saudi Arabia. Most international studies have focused on Western or Asian contexts, with limited emphasis on countries undergoing rapid policy and cultural shifts, as is the case in Saudi Arabia under its Vision 2030. Of the few extant research studies on PA in Saudi Arabia, Abdelhay et al. (8) involved adults (not college-age participants). They focused on sociodemographic and health-related factors, taking into account gender and culture. Aljehani et al. (9) engaged a university-aged demographic, focusing on female participants. This article examines a younger demographic—college-aged men and women in the capital, Riyadh—from a broader perspective on influencing factors. It therefore contributes to filling a gap in the literature.

Furthermore, gender analysis in these transitional periods is often underrepresented. For example, Dambros et al. (19) found that Brazilian female adolescents were significantly less active than males and reported more barriers, particularly related to time, academic pressure, and lack of companionship; challenges that are also repeated among Saudi youth, as noted by Abdelhay et al. (8). Similarly, Lovell et al. (20) demonstrated that female university students in the United Kingdom perceive exertion and fatigue as the primary barriers despite recognizing the benefits of exercise, which highlights a global trend of internal conflict surrounding women's participation in PA.

Additionally, qualitative findings by Anjali and Sabharwal (21) and van Wyk (18) revealed that safety concerns, familial restrictions, and institutional constraints continue to influence young women's experiences, often limiting their access to equitable PA practice opportunities. These issues are consistent with the findings of Aljehani et al. (9), who highlighted how academic load, limited infrastructure, and prevailing gender norms hinder the participation of Saudi female students in sports.

Another relevant gap is the need to explore how emerging adulthood—a developmental stage characterized by identity exploration and autonomy—interacts with these gender-specific barriers. As shown by Hilger-Kolb et al. (22) in Germany and Thomas et al. (23) in Canada, university transitions often result in decreased PA due to disrupted routines, lack of structured opportunities, and time pressure. With its dual pressures of modernization and conservatism, the Saudi context may amplify these transitional vulnerabilities, making research in this area timely and necessary.

Moreover, there is a need to distinguish between internal (e.g., lack of confidence, body image concerns) and external (i.e., environmental safety, access to facilities) barriers. Studies such as those by Rosselli et al. (24) and Shava et al. (25) highlight how psychological and socioeconomic stressors exacerbate perceived barriers and limit engagement. Gender plays a mediating role, as women often face a broader spectrum of deterrents, from cultural expectations to logistical limitations, as reported in He et al. (26) and Espada et al. (27).

By drawing on established methodologies and adapting them to the Saudi context, this descriptive study aims to identify and compare practical, personal, social, environmental, health-related, time-related, and support-related barriers that limit PA participation among college students in Riyadh. It also examines gender-based differences in these barriers to offer actionable insights for promoting more inclusive and equitable participation in PA.

## 2 Methods

### 2.1 Study design

A cross-sectional observational study was conducted in accordance with the STROBE (Strengthening the Reporting of Observational Studies in Epidemiology) guidelines. Ethical standards were maintained in line with the Declaration of Helsinki, and the study protocol was approved by the Institutional Review Board of Prince Sultan University (PSU IRB-2024-11-0201).

### 2.2 Study setting and timeline

The study was conducted in Riyadh between November 24, 2024, and April 30, 2025, using a stratified sampling approach across the city's 15 districts. Participants were recruited from three universities representing different types of institutional ownership: Prince Sultan University (a non-profit institution), Al Yamamah University (a private for-profit university), and Alfaisal University (a non-profit institution). This selection provided representation across different types of higher education institutions in Riyadh. During this period, the two principal investigators, supported by ten research assistants, collected a total of 455 responses from college students across the three participating universities. Of these, 18 were excluded because they did not meet the age eligibility criterion, resulting in a total of 437 valid responses. Data was collected using Google Forms configured to prevent incomplete responses by ensuring all responses were fully completed. After data collection, the dataset was reviewed for accuracy and completeness. Any flagged or inconsistent entries were addressed, and the necessary variables were coded before proceeding with statistical analysis.

### 2.3 Participants

A total of 437 participants completed the questionnaire, comprising 219 females (mean age = 19.19 ± 1.65 years) and 218 males (mean age = 19.51 ± 1.78 years). Inclusion criteria were: being male or female, aged between 18 and 25 years, being enrolled as a college student, and residing in Riyadh. Individuals outside this age range or not residing in Riyadh were excluded. Before participation, all eligible individuals were fully informed about the study's objectives, potential benefits, and associated risks. Written informed consent was obtained from each participant, confirming their voluntary participation in the research.

### 2.4 Data collection instrument and validation process

#### 2.4.1 Questionnaire structure and domains

Since this study relies on a self-administered questionnaire to explore perceived barriers to PA, it was essential to ensure the instrument's validity and reliability prior to conducting the descriptive analysis. Data were collected using a self-administered questionnaire comprising 39 items, provided in Supplementary Material S1. The questionnaire was administered in English to college students enrolled in English-medium instruction programs at the participating universities, with all participants having demonstrated adequate English proficiency through their academic enrollment. The main researches and trained research assistants provided detailed instructions and remained available throughout the administration process to clarify any linguistic ambiguities, ensuring participants fully understood each item before responding. Because the questionnaire was developed and administered in English, and targeted a population fluent in English, a formal translation and back-translation process was not deemed necessary. However, cultural and contextual validity were carefully considered during the instrument development phase. The questionnaire was developed by two research faculty members with extensive academic experience in Saudi Arabia, in collaboration with a team of research assistants—several of whom are native Arabic speakers with in-depth knowledge of the Saudi culture, language, and local context. This collaborative process helped ensure that the items were conceptually clear, culturally appropriate, and contextually relevant for Saudi college students. The development and validation of the questionnaire were conducted as preparatory steps to ensure the accuracy, conceptual clarity, and cultural appropriateness of the data collected for this descriptive study. The instrument included closed-ended, multiple-choice, and numerical response questions organized into nine domains to assess barriers, facilitators, and behavioral aspects related to PA. Each domain targeted a specific construct influencing participation in PA. The domains, number of items per domain, and sample topics are presented in Table 1.

**Table 1:** Thematic domains and corresponding items included in the self-developed questionnaire.

Domain	Items included
Practical Barriers	• Economic access • Domestic demands • Transportation • Availability of facilities • Lack of programs or clubs
Personal Barriers	• Body image • Self-confidence (related to sportswear) • Lack of knowledge and skills in physical activities • Proper equipment • Pressure about social image and masculinization
Social and Cultural Barriers	• Aggressiveness • Competitiveness • Lack of same-gender role models • Media representation • Harassment in PA spaces
Environmental Barriers	• Access to green spaces • Safety • Weather • Environmental pollution • Facility lighting and safety features
Health-Related Barriers	• Health conditions • Fear of injury • Fatigue • Physical pain • Recovery from injuries or previous conditions
Social Support	• Lack of family support • Lack of friend support • Lack of activity group support • Lack of network support (University/work peers) • Lack of coach or instructor support
Time Constraints	• Lack of time (family responsibilities) • Lack of time (academic demands) • Lack of time (work obligations) • Other priorities • Time optimization
PA Engagement	• Weekly frequency of PA • Type of PA practice
Personal Data	• Age • Gender

Each domain contained five items, grouped according to theoretical coherence and informed by empirical literature and the contextual characteristics of the Saudi environment. Most items were rated on a five-point Likert scale (1–5), where higher scores reflected fewer perceived barriers or greater support for PA. Exceptions included demographic items (e.g., age, gender) and questions on frequency and type of PA, which used categorical or numerical formats. The participation in physical activity was assessed through two self-reported items designed to capture weekly frequency and type of practice. The first item (“How often do you engage in physical activity each week?”) offered options ranging from 0 times per week (no practice) to 7 days per week. The second item (“How would you describe your level of participation in physical activity?”) classified practice into five categories: no practice, occasional practice (recreational), regular practice (informal), consistent participation in organized physical activities, or federated (competitive) practice. The full questionnaire is available in Supplementary Material S1. PA was defined as any bodily movement produced by skeletal muscles that results in energy expenditure (28). This comprehensive structure was intended to capture a multidimensional and ecological understanding of PA behavior, incorporating motivational, environmental, and social factors in alignment with theoretical models such as the Social Ecological Model and Self-Determination Theory (29). Domain scores were calculated as the mean of the five Likert-scale items per domain (range: 1–5). A higher average score indicates fewer perceived barriers or greater facilitators within that domain.

#### 2.4.2 Instrument development and content validation

The questionnaire was developed following a structured validation process overseen by a panel of five sports science experts. The panel initially defined and evaluated the relevance of the study domains using a 0–10 rating scale. Individual items were then formulated for each domain and assessed by the experts. To quantify content validity, the Content Validity Index (CVI) for each domain (D) and item (I) was calculated using the formula D/I-CVI = Ne/Nt, where Ne represents the number of experts rating the item as “very relevant” and Nt the total number of experts. Items and domains with a CVI above 0.80 were retained in the final questionnaire, ensuring high levels of relevance and clarity. An exploratory factor analysis (EFA) was conducted separately for male and female participants to assess construct validity. The Kaiser–Meyer–Olkin (KMO) measure yielded excellent values (0.952 for males and 0.943 for females), and Bartlett's test of sphericity was significant in both groups (*p* < 0.001), confirming the suitability of the data for factor extraction. These procedures were not intended as the primary focus of the study, but rather as essential preparatory steps to ensure the quality and appropriateness of the data used for the subsequent descriptive analysis.

#### 2.4.3 Convergent validity

To assess convergent validity**,** the self-developed instrument was compared with a previously validated questionnaire: the *Plateful of Prevention (PoP)* tool, created by Oregon State University in collaboration with the USDA and CDC (Table 2). The PoP includes items showing conceptual or partial equivalence with those in the present questionnaire (30). Given the differences in scale structure—the PoP employs a four-point Likert scale (0–3), and the current instrument uses a five-point Likert scale (1–5) with reversed scoring—PoP items were adapted by converting them to a five-point format and reversing their scoring direction to align scales for comparability. Based on item-level mappings, all corresponding item pairs showed Pearson correlation coefficients exceeding 0.75, indicating a strong relationship and confirming convergent validity (Table 2). This analysis demonstrated that, despite including original dimensions tailored to the Saudi context (e.g., academic time demands, perceptions of physical education structure), the instrument retains conceptual overlap with established measurement tools. The adapted content from the PoP tool was used in accordance with the public domain guidelines provided by the Centers for Disease Control and Prevention (CDC), which state that most materials on CDC and USDA websites may be reused without permission, provided they are properly cited and do not implied endorsement. Accordingly, the adapted items were used solely for academic purposes to assess construct alignment and convergent validity.

**Table 2:** Item mapping between the self-developed questionnaire and the PoP instrument for convergent validity analysis.

Item of the self-administered questionnaire	Topic	PoP item	Topic	Correlation
7. Lack of time (Family responsibilities)	Time constrains-family	8. PA takes too much time…	General time constraints	0.75
14. Lack of time (Academic demands)	Time constrains-academic	1. My day is so busy…	Lack of time	0.81
21. Lack of time (Work obligations)	Work time	3. I'm too tired after work…	Work-related fatigue	0.77
35. Time optimization	Time Management	1. My day is so busy…	Lack of time	0.82
2. Body image	Body Image	9. I'm embarrassed how I look…	Body image	0.79
16. Knowledge and skills in physical activities	Lack of Knowledge and skills	6. I've never learned skills…	Lack of skills	0.85
4. Access to green spaces	Environment	7. I don't have access…	Lack of infrastructure	0.89
19. Fatigue	Fatigue	3. I'm too tired after work…	Tiredness	0.87
6. Lack of family support	*Social Support*	2. None of my family members…	Family support	0.86

#### 2.4.4 Instrument reliability

To ensure the reliability of the self-administered questionnaire, two types of analyses were conducted: internal consistency, assessed through Cronbach's alpha coefficient, and temporal reliability, evaluated using the test-retest method (Table 3), with a 14-day interval between administrations. The Cronbach's alpha coefficients obtained for each domain indicated high internal consistency, with values ranging from 0.83 to 0.91. This suggests that the items within each dimension are strongly correlated and consistently measure the intended construct. All values exceeded the commonly accepted threshold of 0.70 (31), supporting the strong internal reliability of the instrument.

**Table 3:** Cronbach's alpha coefficients and test-retest reliability for each domain.

Domain	Pearson's r test-retest interval (14 days)	Cronbach's alpha (Internal consistency)
Practical Barriers	0.82	0.85
Personal Barriers	0.85	0.90
Social and Cultural Barriers	0.80	0.85
Environmental Barriers	0.89	0.90
Health-Related Barriers	0.83	0.88
Social Support	0.86	0.91
Time Constraints	0.88	0.91
PA Engagement	0.78	0.83

Temporal reliability was measured using Pearson's correlation coefficient (r) on a subsample of 56 subjects (29 females and 27 males) who completed the questionnaire on two separate occasions, 14 days apart. The resulting coefficients showed high temporal stability across all dimensions, with values between 0.78 and 0.89 (Table 3). These findings indicate that the instrument provides consistent results over time and is not significantly affected by short-term or situational factors. These results reinforce the psychometric soundness of the questionnaire and support its use as a reliable measurement tool in the context of this study. Similar instruments have been used in previous research on PA behaviors, health promotion, and educational assessment, contributing to the growing number of standardized tools in these fields. Therefore, the questionnaire is considered appropriate for evaluating the variables of interest in college students.

#### 2.4.5 Justification for instrument design

Although various validated instruments exist to assess specific PA barriers and facilitators, developing a context-sensitive and customized questionnaire was necessary to address the unique cultural, institutional, and curricular factors affecting Saudi college students. Existing tools often omit emergent contextual variables, such as peer dynamics in physical education classes or availability and perception of sports-related educational resources (9). The developed instrument successfully integrates motivational, environmental, and social dimensions into a unified and comprehensive measure, providing an empirically supported tool with strong internal consistency and convergent validity. Thus, it fills a relevant gap in the literature and offers a practical contribution to evaluating PA determinants within this population (32).

### 2.5 Bias mitigation strategies

To minimize potential bias in this study, a stratified sampling method was implemented in Riyadh's 15 districts to ensure proportional representation of the diverse university-aged population. This approach helped reduce selection bias and ensure adequate representation of different geographic and demographic subgroups. However, it must be acknowledged that the sample might not fully represent individuals from regions beyond the Saudi capital. To mitigate response bias, participant anonymity was guaranteed, and the voluntary and confidential nature of the questionnaire was emphasized. This encouraged honest and thoughtful responses. The research team supervised the administration of the questionnaire in person, providing instructions and clarifying questions to minimize misunderstandings, which also helped reduce response errors. Furthermore, the use of Google Forms with required fields prevented incomplete submissions, further improving data integrity and reliability.

The questionnaire was carefully developed and validated with cultural relevance for the Saudi context to address measurement bias, including expert review and factor analysis. The instrument's internal consistency and test-retest reliability further minimized measurement errors. Consistent questionnaire administration procedures across all participants helped reduce information bias by standardizing data collection methods. Finally, the principal investigators thoroughly reviewed the dataset to identify and resolve any marked or inconsistent responses prior to analysis. Descriptive statistics revealed no unusual response patterns or skewness, supporting the integrity and validity of the collected data.

### 2.6 Sample size determination

To calculate the sample size, the following formula was employed: *n* = *Z*^2^.*p*.*q*.*N*/*e*^2^.(*N* − 1)+*Z*^2^.*p*.*q*. This formula incorporates key variables such as sample size (*n*), confidence level (*Z*), probability of success (*p*), population size (*N*), probability of failure (*q*), and confidence interval (*e*) (33). Within the scope of this research, a 95% confidence level (corresponding to a Z-value of 1.96), a 5% margin of error, and an estimated population proportion (*p*) of 50% were chosen. Given these criteria, a minimum of 385 participants was established to secure a representative sample. While the sample size was calculated using a single-proportion formula, the final sample included nearly equal numbers of males and females, which allowed for adequately powered gender comparisons.

### 2.7 Statistical analysis

The statistical analysis was conducted using IBM SPSS Statistics software (Version 26.0). Descriptive statistics are presented as means and standard deviations $\left(\bar{X}\pm \mathrm{SD}\right)$. The Kolmogorov–Smirnov test was applied to assess the normality of continuous variables, and Levene's test was used to verify the assumption of homoscedasticity. To compare PA engagement and perceived barriers between male and female college students, independent samples *t*-tests were performed for each barrier and for weekly frequency and type of PA. Effect sizes were calculated using Cohen's d, with values of 0.2, 0.5, and 0.8 interpreted as small, medium, and large effects, respectively (34). A multiple linear regression analysis was conducted to examine whether individual items from the perceived barrier domains, along with gender and age, could significantly predict the weekly frequency and type of PA. To minimize the risk of multicollinearity—particularly given the conceptual overlap among items within the same domain—several steps were taken. The Variance Inflation Factor (VIF) was calculated for each predictor, and all values were below the commonly accepted threshold of 5, with none exceeding 2. Therefore, no items were removed or combined due to concerns about multicollinearity. Additionally, a stepwise selection method was applied to retain only the most significant and non-collinear predictors, thereby improving the stability and interpretability of the model. All standard regression assumptions—including the normal distribution of residuals, absence of multicollinearity, and homoscedasticity—were verified and satisfied prior to conducting the analysis. The level of statistical significance was set at *p* < 0.05.

## 3 Results

**Figure 1:**
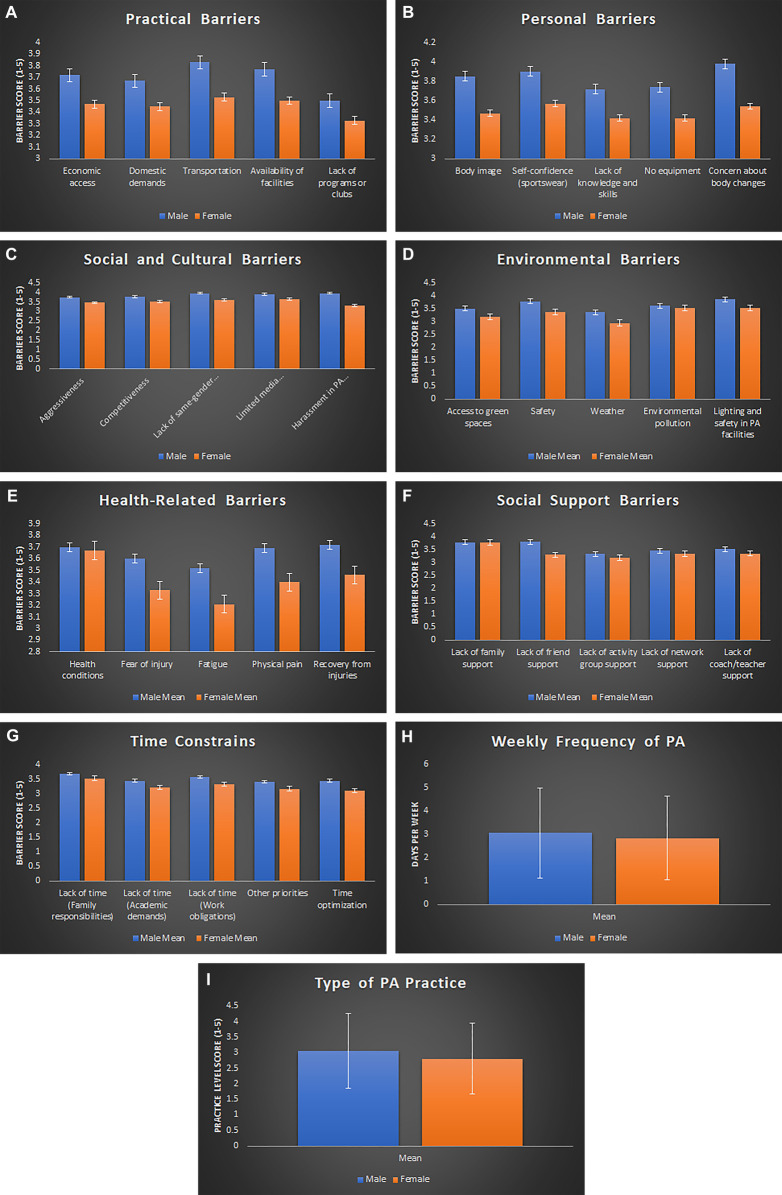
A composite figure presents the survey results on physical activity (PA) barriers and engagement, separated by gender. Panels **A–G** display mean scores on a 1–5 Likert scale, with higher scores indicating a lower perception of barriers. The figure shows Panel **A** representing practical barriers, which include economic access, domestic demands, transportation, availability of facilities, and lack of programs or clubs. Panel **B** shows personal barriers, including body image, self-confidence related to sportswear, lack of knowledge and skills, no equipment, and concern about body changes. Panel **C** highlights social and cultural barriers, such as aggressiveness, competitiveness, lack of same-gender role models, limited media representation, and harassment in PA spaces. Panel **D** presents environmental barriers, including access to green spaces, safety, weather, environmental pollution, and lighting. Panel **E** displays health-related barriers, which include health conditions, fear of injury, fatigue, physical pain, and recovery from injuries or previous conditions. Panel **F** focuses on social support barriers, including lack of family support, friend support, activity group support, network support (university/work peers), and coach/teacher support. Panel **G** represents time constraints, including family responsibilities, academic demands, work obligations, other priorities, and time optimization. Panel **H** presents the weekly frequency of PA, measured in days. Panel **I** presents the type of PA practice, ranging from 1 (no practice) to 5 (competitive level). Error bars represent the standard error of the mean.

After confirming normality and homoscedasticity, independent samples *t*-tests were conducted to compare male and female participants across all measured items. The analysis revealed statistically significant gender differences in multiple perceived barriers to PA, as summarized in Table 4 and illustrated in Figures 1A–I. The items are listed below in descending order of effect size: *Harassment in PA spaces, lack of friend support, concern about body changes, safety, body image, weather, lack of same-gender role models, lighting and safety in PA facilities, time optimization, self-confidence (sportswear), no equipment, access to green spaces, physical pain, lack of knowledge and skills in physical activities, aggressiveness, fatigue, transportation, type of PA practice, limited media representation by gender, availability of facilities, fear of injury, recovery from injuries or previous conditions*.

**Table 4:** Gender differences in perceived barriers to PA: descriptive statistics and T-test results by item.

Domain	Items included	Male	Female	T-test *p*-value	Cohen's d
Practical Barriers	Economic access	3.72 ± 1.41	3.47 ± 1.32	0.056	0.178
	Domestic demands	3.67 ± 1.34	3.45 ± 1.31	0.095	0.158
	Transportation	3.83 ± 1.40^a^	3.53 ± 1.37	0.024	0.215
	Availability of facilities	3.77 ± 1.31^a^	3.50 ± 1.33	0.034	0.204
	Lack of programs or clubs	3.50 ± 1.38	3.33 ± 1.31	0.196	0.124
Personal Barriers	Body image	3.85 ± 1.30^a^	3.47 ± 1.32	0.002	0.296
	Self-confidence **(**sportswear)	3.90 ± 1.36^a^	3.57 ± 1.34	0.010	0.248
	Lack of knowledge and skills in physical activities	3.72 ± 1.37^a^	3.42 ± 1.32	0.018	0.227
	No equipment	3.74 ± 1.37^a^	3.42 ± 1.23	0.010	0.247
	Concern about body changes	3.98 ± 1.32^a^	3.54 ± 1.33	0.001	0.328
Social and Cultural Barriers	Aggressiveness	3.75 ± 1.33^a^	3.46 ± 1.33	0.021	0.222
	Competitiveness	3.77 ± 1.35	3.52 ± 1.28	0.052	0.187
	Lack of same-gender role models	3.96 ± 1.26^a^	3.61 ± 1.29	0.004	0.280
	Limited media representation by gender	3.90 ± 1.31^a^	3.63 ± 1.22	0.027	0.211
	Harassment in PA spaces	3.95 ± 1.32^a^	3.31 ± 1.28	<0.001	0.493
Environmental Barriers	Access to green spaces	3.50 ± 1.40^a^	3.17 ± 1.30	0.013	0.239
	Safety	3.78 ± 1.31^a^	3.37 ± 1.26	0.001	0.313
	Weather	3.36 ± 1.41^a^	2.95 ± 1.39	0.002	0.291
	Environmental pollution	3.61 ± 1.38	3.52 ± 1.29	0.440	0.074
	Lighting and safety in PA facilities	3.86 ± 1.25^a^	3.52 ± 1.30	0.005	0.268
Health-Related Barriers	Health conditions	3.70 ± 1.40	3.67 ± 1.27	0.811	0.020
	Fear of injury	3.60 ± 1.36^a^	3.33 ± 1.34	0.038	0.199
	Fatigue	3.52 ± 1.41^a^	3.21 ± 1.37	0.023	0.219
	Physical pain	3.69 ± 1.27^a^	3.40 ± 1.29	0.017	0.232
	Recovery from injuries or previous conditions	3.72 ± 1.34^a^	3.46 ± 1.39	0.045	0.196
Social Support	Lack of family support	3.78 ± 1.39	3.77 ± 1.30	0.950	0.006
	Lack of friend support	3.80 ± 1.37^a^	3.30 ± 1.37	<0.001	0.367
	Lack of activity group support	3.33 ± 1.51	3.18 ± 1.47	0.286	0.101
	Lack of network support (University/work peers)	3.45 ± 1.44	3.34 ± 1.36	0.387	0.081
	Lack of coach/teacher support	3.52 ± 1.48	3.35 ± 1.39	0.225	0.113
Time Constraints	Lack of time (Family responsibilities)	3.69 ± 1.32	3.53 ± 1.26	0.176	0.127
	Lack of time (Academic demands)	3.45 ± 1.40	3.21 ± 1.40	0.069	0.174
	Lack of time (Work obligations)	3.58 ± 1.40	3.32 ± 1.34	0.053	0.182
	Other priorities	3.41 ± 1.28	3.17 ± 1.27	0.055	0.183
	Time optimization	3.45 ± 1.33^a^	3.10 ± 1.36	0.007	0.262
PA Engagement	Weekly frequency of PA	3.06 ± 1.94	2.84 ± 1.80	0.212	0.115
	Type of PA Practice	3.06 ± 1.21^a^	2.80 ± 1.14	0.021	0.214

^a^ Significant difference found between males and females (*p* < 0.05).

In contrast, no statistically significant gender differences were found in the following items: *Competitiveness, other priorities, lack of time (work obligations), economic access, lack of time (academic demands), domestic demands, lack of time (family responsibilities), lack of programs or clubs, weekly frequency of PA, lack of coach/teacher support, lack of activity group support, lack of network support (university/work peers), environmental pollution health conditions, and lack of family support*.

Regarding PA frequency, the stepwise multiple linear regression analysis (Table 5), provided a final model that included four significant predictors, listed in decreasing order of importance based on their standardized coefficients (*β*): *Other Priorities, Weather, Transportation,* and *Fatigue.* All four were statistically significant negative predictors, suggesting that a higher perception of these barriers is associated with a lower frequency of PA practice. Multicollinearity diagnostics indicated no concerns, with all Variance Inflation Factor (VIF) values below 2.

**Table 5:** Stepwise multiple linear regression predicting weekly PA frequency from perceived barriers.

R^2^	p-Value (model)	Dependent variable	Independent variables	Standardized coefficient (*β*)	*p*-Value (variable)
0.144	0.03	Weekly PA frequency	Other Priorities	−0.276	0.030
			Weather	−0.167	0.032
			Transportation	−0.120	<.001
			Fatigue	−0.117	<.001

In terms of the type of PA practice (Table 6), the regression analysis yielded a final model comprising five significant predictors: *Environmental pollution, lack of same-gender role models, time optimization, lack of activity group support,* and *lack of knowledge and skills in physical activities*, also listed in order of importance according to their *β* values. All predictors were statistically significant and negatively associated with the type of PA practice, indicating that greater perception of these barriers is linked to reduced participation in more structured or organized forms of PA. Again, multicollinearity was not an issue, as VIF values ranged from 1.463 to 1.930, well within acceptable limits.

**Table 6:** Stepwise multiple linear regression predicting type of PA practice from perceived barriers.

R^2^	*p*-Value (model)	Dependent variable	Independent variables	Standardized coefficient (β)	*p*-value (variable)
0.177	<.001	Type of PA practice	Environmental pollution	−0.683	<.001
			Lack of same-gender role models	−0.569	<.001
			Time optimization	−0.563	<.001
			Lack of activity group support	−0.559	<.001
			Lack of knowledge and skills in PA	−0.518	<.001

## 4 Discussion

This descriptive study identified and compared key perceived barriers to PA among college students in Riyadh, highlighting significant gender-based differences across multiple domains to inform targeted public health strategies. The most striking finding was that Personal Barriers exhibited the highest number of significant gender differences, with all five items showing statistical variation. This was followed closely by Social and Cultural, Environmental, and Health-Related Barriers, each with four of five items differing significantly. Practical Barriers accounted for two of five, while Social Support and Time Constraints showed only one significant difference each. Overall, 21 out of 35 items revealed statistically significant gender differences, with females consistently reporting higher levels of perceived barriers than males. However, it is important to note that most of these differences demonstrated small effect sizes, with only harassment in PA spaces showing an effect size close to medium. While statistically significant, these modest effect magnitudes suggest that gender differences should be interpreted with caution when developing policy interventions, as their real-world impact may vary across different cultural and institutional contexts. These results are in line with previous research, where female students often report more intense and numerous PA barriers than their male counterparts (8, 9, 17, 24). Such gender-specific disparities have been especially notable in personal and social domains across a range of cultural contexts, including those of Saudi Arabia, India, and various European countries (6, 20, 35). These consistent findings may reflect the interplay between sociocultural expectations, gender norms, and institutional limitations that differentially shape how men and women experience opportunities for PA.

Regarding Personal Barriers, gender divergence was particularly pronounced, which underscores the psychological and sociocultural pressures that influence women's participation in PA. A key issue was self-confidence related to sportswear, as many women reported discomfort when wearing PA-appropriate clothing, especially in mixed-gender or public settings. This discomfort often stems from heightened self-consciousness and fear of judgment, rooted in societal norms that emphasize modesty and discourage physical exposure (36). Such concerns can inhibit participation even when interest and motivation are present. Similarly, Aljehani et al. highlighted sociocultural barriers such as gender roles and adherence to cultural standards as significant factors limiting female university students' physical activity in Saudi Arabia (9).

Despite the statistical significance of these personal barriers, the effect sizes were generally small to medium, suggesting that while these concerns are real and consistent, their individual impact may be more manageable through targeted interventions than initially apparent.

Closely linked to this is the persistent problem of body image, where societal expectations impose beauty ideals that are difficult to achieve or unrealistic. Women often internalize negative self-perceptions, especially when engaging in PA in environments where their bodies may be scrutinized. This discomfort is magnified by media and cultural narratives that equate female beauty with slenderness rather than physical strength. As noted by Brown & Bowmer, aesthetic concerns are powerful deterrents for young women, especially in societies where female appearance is subject to intense scrutiny (37). Similarly, Martínez-Sánchez et al. found that body image concerns among female university students were closely linked to lower mental well-being, illustrating the emotional toll these ideals impose (38).

In addition to general body image issues, many participants expressed concern over body image changes resulting from PA, particularly the fear of developing muscularity—an attribute often perceived as unfeminine in traditional cultural contexts. In societies where thinness and delicacy are idealized, strength-based or high-intensity exercise may be seen as incompatible with prevailing beauty standards. This internalized pressure can lead women to avoid certain types of PA despite personal interest or health benefits. These dynamics are consistent with the findings of Brown & Bowmer and Aguirre Chavez et al., who describe body image as a central concern influencing women's decisions around PA engagement (37, 39). This highlights how internalized gender ideals can directly suppress behavior that would otherwise align with health promotion goals, suggesting a conflict between personal agency and sociocultural conformity. Although these body image-related barriers showed small to medium effect sizes, their cumulative impact and cultural entrenchment suggest they require sustained, multi-level interventions rather than singular policy approaches.

Another frequently cited barrier was lack of knowledge and skills in physical activities. Many female participants reported feeling unprepared or unsure about how to engage in exercise properly, which in turn diminished their motivation and confidence. This gap in practical competence can discourage trial and repetition—two critical components for habit formation in PA. As Khan et al. highlight, self-efficacy is crucial for overcoming initial hesitation, and when lacking, it can contribute to long-term avoidance (40). This also implies that interventions targeting skill acquisition and familiarity may be more effective than motivational campaigns alone.

The issue of no equipment also emerged as a practical limitation that particularly affects women. Limited access to appropriate gear—whether due to financial constraints, cultural restrictions, or availability—reduces opportunities for engagement in structured or skill-based activities. In some cases, this lack of equipment is exacerbated by gendered disparities in support from family or peers, further widening the participation gap.

Turning to Social and Cultural Barriers, four of the five items also showed significant gender differences. These barriers are deeply embedded in the social norms, safety perceptions, and cultural expectations that shape female behavior in the context of PA in Riyadh. Among the most reported challenges was the experience or fear of harassment in PA spaces. In a society where gender segregation and modesty are strongly emphasized, public or mixed-gender exercise environments may evoke discomfort or fear for women (9). The lack of accessible, female-only facilities exacerbates this vulnerability. Almahmood et al. noted that many Saudi women prefer enclosed spaces like shopping malls for walking, as outdoor areas tend to be male-dominated (41). This spatial patterning of safety and exposure reveals how cultural constructions of space and gender intersect to constrain female mobility and discourage PA. Similar patterns were highlighted by Khan et al., van Wyk, and He et al., who each observed how traditional gender roles and safety concerns shape women's experiences in sport and exercise contexts (18, 26, 40). Interestingly, Frühauf et al. found that women who participated in less gender-conforming sports reported fewer harassment-related barriers, highlighting the moderating effect of community norms and subcultural spaces (42). This suggests that fostering supportive subcultures around PA may help mitigate some of the more rigid mainstream gender constraints.

The absence of same-gender role models was another prominent barrier, reflecting a symbolic limitation that reinforces material constraints. When women do not see themselves represented in sports leadership, coaching, or participation, it becomes difficult to imagine themselves in these roles. The motivational and aspirational power of role models is undermined by their scarcity, particularly in cultures where female athleticism is discouraged. Van Wyk and Frühauf et al. emphasized that visibility—whether through coaches, athletes, or peers—can reshape participation trends (18, 42). Similarly, He et al. observed that enduring gender stereotypes depicting women as weak or passive discourage many from pursuing athletic pursuits, unless these narratives are explicitly countered (26). This invisibility contributes to a cycle of exclusion, where the lack of representation reduces participation, which in turn sustains underrepresentation. Although the gender differences in these barriers reached statistical significance, their effect sizes were modest. These results underscore important gender-related patterns but also highlight the need for context-sensitive policies and programs, rather than broad, one-size-fits-all approaches.

Closely related to this is the perception of aggressiveness in PA environments. Competitive, intense, or male-dominated exercise settings can feel exclusionary or intimidating to women, especially those without a background in team sports or structured activity. Such environments may reinforce the notion that PA is a male space, where women must adapt to masculine norms to participate. For many, this can be a source of discomfort and alienation. Frühauf et al. observed that women tend to prefer cooperative, community-based forms of exercise, suggesting that overly aggressive or competitive cultures may function more as barriers than motivators for female participation (42). This suggests a need to reconceptualize PA environments in ways that prioritize inclusivity and flexibility, rather than competitiveness alone.

Finally, the media exacerbates these cultural challenges. The lack of active women in advertising, television, and sports leads many to believe that PA is not a suitable option for them. The message is clear: if women are not visible, they do not belong. Van Wyk argued that media representation functions not just as inclusion but as affirmation (18), while He et al. demonstrated that media silence around female athleticism sustains harmful stereotypes (26). In sum, these findings reveal how layered cultural norms—through fear, aesthetics, visibility, and exclusion—interact to create a powerful system of constraints that limit women's opportunities and willingness to engage in PA. Addressing these will require not only safe and inclusive facilities, but also broader cultural and media reforms that reshape how society views and values female physicality.

The impact of these barriers is further intensified by a lack of social support, particularly from peers. The absence of friend support emerged as a gendered challenge, pointing to the importance of interpersonal networks in sustaining motivation for PA. For many female students, peer encouragement may be limited in environments that prioritize academic or domestic responsibilities over physical health. Additionally, the lack of female-only group activities can restrict opportunities for shared motivation and community building. Given the strong influence of social connection on long-term engagement, this lack of peer reinforcement becomes a critical obstacle. Hilger-Kolb et al. and Shava et al. also identified peer disengagement as a prominent barrier for female students (22, 25), while Lovell et al. and Brown & Bowmer noted that collective motivation—particularly from peers—is often more influential for women than individual drive (20, 37). Thus, the lack of social support reveals not only structural gaps but also deeper social dynamics that continue to inhibit women's full participation in PA. These findings further suggest that social interventions—such as peer-led PA programs—may be as critical as infrastructural changes in addressing gender disparities in PA.

Beyond social dynamics, structural limitations play a critical role in shaping gendered access to PA. Regarding Practical Barriers, two obstacles stood out clearly: lack of transportation and the availability of facilities. Transportation emerged as a significant challenge for female participants, reflecting ongoing restrictions on women's mobility and independence. In Riyadh, factors such as cultural expectations, family dynamics, and infrastructure can limit women's freedom of movement, especially when they rely on others to access exercise locations (43). This logistical dependency makes planning and maintaining regular PA routines more difficult, reinforcing the perception that PA is less accessible for them. Aljehani et al. and Anjali & Sabharwal also identified transportation as a major barrier for female students in contexts with limited mobility options (9, 21). In addition, the availability of adequate facilities posed a substantial challenge. Even when women manage to reach exercise centers, the available options are often fewer, less adequately equipped, or poorly aligned with their schedules and comfort levels. Many women-only gyms operate under restricted hours, offer limited space, or charge fees that are unaffordable for university students. The perception that exercise environments are unwelcoming or impractical further discourages participation. These findings reinforce the idea, supported by Aljehani et al. and Anjali & Sabharwal, that access to suitable facilities represents a structural gender barrier that marginalizes female participation in PA contexts (9, 21). Taken together, these barriers highlight the need for more inclusive physical spaces, improved infrastructure, and transportation systems that actively support women's participation. Even the most motivated individuals may be constrained by logistical realities that are gendered in origin.

Environmental factors also substantially influence perceived barriers, especially from a gendered perspective. Within the domain of Environmental Barriers, safety concerns were particularly prominent among female participants. Many women reported feeling unsafe exercising outdoors or in unsupervised facilities, especially in the absence of female-only spaces. This sense of vulnerability—intensified during early morning or evening hours—discourages participation and reinforces the association between public PA and personal risk. These results align with findings from Anjali & Sabharwal, who identified safety as a key barrier among female students in India (21), and Aljehani et al., who noted how cultural norms and infrastructure deficits in Saudi Arabia exacerbate such concerns (9).

Environmental discomfort is further compounded by Riyadh's extreme climate, particularly its intense heat. While high temperatures affect everyone, their impact might be greater for women due to cultural dress requirements. Wearing abayas or hijabs could increase physical discomfort in extreme heat, reducing the feasibility of outdoor exercise. This gender dynamic mirrors findings from Pandolfo et al. and Dambros et al., who reported similar difficulties among adolescent girls in hot climates (19, 44). Another environmental concern was the lighting and supervision of exercise facilities. Women were more affected by poor lighting or a lack of oversight in gyms, which triggered safety concerns—particularly when they already face greater vulnerability in public spaces. These factors, often overlooked in public policy, play a decisive role in everyday decisions around PA. Hilger-Kolb et al. highlighted the importance of infrastructure and transition spaces, showing how perceived risk directly impacts female participation (22). Limited access to green spaces was another obstacle reported by participants. Although parks and recreational areas are theoretically open to all, they may be perceived as male-dominated or culturally inappropriate for women—especially in the absence of segregated areas or privacy measures. This perception discourages use and limits opportunities for spontaneous or informal PA. Research by Hilger-Kolb et al. and Frühauf et al. emphasizes how inclusive urban planning can either facilitate or hinder PA, demonstrating that perceptions of safety and social appropriateness deeply influence women's access to such spaces (22, 42). Addressing these concerns requires gender-sensitive urban and architectural planning that considers not only physical infrastructure but also the social and symbolic dimensions shaping women's embodied experiences in public spaces.

Unlike other domains, only one item under time-related barriers showed a significant gender difference: Time Optimization, with women reporting greater difficulty managing time for PA. This finding aligns with previous studies (Justine et al., 3; Hoare et al., 16; Hasan et al., 35), where women typically report more time-related barriers due to domestic and caregiving responsibilities (3, 16, 35). In the Saudi context, this may reflect traditional gender role expectations where young women often have additional family and household responsibilities that create more complex time management challenges for incorporating PA into their daily routines.

In the domain of Health-Related Barriers, women again reported higher scores on multiple items, indicating greater sensitivity to physical or physiological limitations. Physical pain stood out as a frequent obstacle, possibly linked to menstrual discomfort, lower baseline fitness levels, or fears associated with exertion. These findings align with Dambros et al. and Khan et al., who found that pain and physical discomfort discourage women more from maintaining a regular exercise routine (19, 40). Fatigue also emerged as a significant barrier. Beyond physiological differences, women often face the combined burden of academic, social, and domestic demands, which depletes their energy and causes PA to be perceived as an additional strain. This pattern is consistent with Justine et al. and Hoare et al., who highlighted the close link between life stressors and lower PA levels (3, 16). Concerns related to physical pain and recovery from injuries or previous conditions were more pronounced among women, reflecting both psychological and structural barriers that hinder sustained PA participation. Women with limited prior training or low confidence in technique may be more apprehensive about sustaining injuries, particularly when access to proper instruction, supervision, or recovery support is lacking. This fear is often exacerbated by unfamiliarity with equipment or absence of gender-sensitive environments. As noted by Dambros et al. and Khan et al., fear of injury operates as a significant psychological barrier, particularly among women with limited exposure to formal exercise contexts (19, 40). In parallel, recovery from previous injuries or conditions can itself become a prolonged obstacle. Female participants may feel underserved by rehabilitation systems, especially when access to female physiotherapists or trainers is limited, or when recovery settings are not culturally or socially accommodating. These concerns can delay or prevent resumption of PA. This suggests that perceived physical vulnerability is not only biological but also socially constructed, shaped by limited access to safe and supportive recovery environments. Addressing these gaps requires not only improved services but also greater normalization of female physical resilience. Both Anjali & Sabharwal and Aljehani et al. emphasize the need for gender-responsive rehabilitation strategies and institutional support to reduce these compounded barriers and promote safe, consistent re-engagement in PA (9, 21).

In terms of actual PA engagement, a clear gender difference emerged in activity preferences, despite similar weekly frequency. Both men and women reported a comparable number of sessions per week. However, men tended to gravitate toward organized or competitive sports—such as university teams—while women favored recreational or informal activities. This difference reflects broader sociocultural influences that shape how individuals engage with PA. For men, fewer perceived barriers across personal, social, and environmental domains facilitate access to structured athletic environments—a dynamic reinforced from a young age. This aligns with Frühauf et al. and van Wyk, who emphasized the role of early socialization and male access to formal athletic opportunities (18, 42). In contrast, women face multiple obstacles—harassment in PA spaces, lack of peer support, body image pressures, safety concerns, and a shortage of female role models—that deter participation in public or competitive settings. Consequently, they tend to engage more in private or informal activities such as walking, home workouts, or small group sessions with friends Morris et al., (45). Although frequency may appear similar, the contexts and social dynamics of participation differ significantly, influencing motivation, access, and sustainability. This distinction is crucial: while metrics like frequency suggest parity, they obscure deeper inequities related to autonomy, safety, and cultural acceptance that underpin women's participation. This pattern was also noted by Hilger-Kolb et al. and Justine et al., who found informal PA often represents a more practical and attractive option for women facing unique barrier (16, 22). Recovery after injury was another area where women reported more difficulties. Limited support from institutions and concerns about getting injured again in mixed-gender settings may cause delays or even prevent women from returning to exercise. These issues highlight the need for rehabilitation services tailored to women's specific cultural and social contexts (Anjali & Sabharwal,; Aljehani et al.) (9, 21).

As a positive note, the effect sizes for gender differences in perceived barriers to PA were generally small. This means that while differences are present, they can be addressed. Since these differences are not large, targeted efforts may be especially effective. With adequate policies, inclusive programs, and cultural modifications, disparities in participation styles and access could be reduced—promoting more equitable engagement over time. This optimistic perspective aligns with Martínez-Sánchez et al. and Anjali & Sabharwal, who advocate for gender-sensitive interventions and higher efforts in society to improve equality in PA (21, 38).

Additionally, this study's regression analyses offered deeper insights into the perceived barriers that predict PA behavior, complementing earlier gender-based findings. Regarding weekly PA frequency, significant negative predictors included barriers related to Competing Priorities, Weather, Transportation, and Fatigue. This indicates that individuals who perceive these barriers more intensely tend to be less active, highlighting how time management and environmental conditions affect daily life—impacting both genders, albeit with unique implications in Riyadh's context. In this setting, where gender roles are more rigidly defined, logistical constraints take on different meanings: for men, they may relate to productivity and ambition; for women, they are often linked to permission, safety, and access—suggesting that even shared barriers are experienced through a gendered lens. In contrast, predictors for PA type revealed different factors: Environmental Pollution, Lack of Same-Gender Role Models, Time Optimization, Lack of Group Support for PA, and Lack of Knowledge or Skills in PA—all were negatively associated with participation in structured or organized activities. This reinforces the idea that beyond frequency, the quality and context of participation are shaped by social and structural barriers—many of which disproportionately affect women, as previously discussed. It is important to acknowledge, however, that the predictive power of these models was modest, with R^2^ values of 0.144 for PA frequency and 0.177 for PA type. This indicates that while the perceived barriers measured in this study—including practical, personal, social, cultural, environmental, health-related, and time constraints—are relevant, they explain only a small portion of the variance in PA behavior. This suggests that PA engagement is influenced by additional factors or complex interactions not fully captured by these measures. The multifaceted nature of PA behaviors calls for further research employing broader or complementary approaches to better understand and predict participation patterns. Therefore, effective interventions need to address both logistical barriers (e.g., transportation, time, environmental conditions) and sociocultural factors (e.g., gender representation, support networks). This approach aligns with the recommendations of Martínez-Sánchez et al. and Hilger-Kolb et al., who highlight the importance of considering environmental, social, and individual factors to reduce barriers and increase gender-equitable participation in PA (22, 38).

Although this study provides valuable insights into perceived barriers to PA among university youth in Riyadh, several limitations must be acknowledged. First, the cross-sectional design limits the ability to draw causal inferences; associations between perceived barriers and PA behaviors are correlational, not directional. Longitudinal designs would offer better insight into how these barriers develop and influence sustained participation over time. Second, although gender comparisons were central to the study's aims, the sample size was calculated using a single-proportion formula rather than a comparative two-proportion approach. However, the final sample included nearly equal numbers of males and females, which allowed for reasonably powered comparisons. Future studies should employ sample size estimation methods appropriate for group-based hypotheses to enhance statistical rigor. Third, the study did not consider seasonal variation, which may influence PA behaviors and perceived barriers—particularly in regions with extreme weather conditions such as Riyadh. Future research should adopt longitudinal or repeated cross-sectional approaches to better capture seasonal effects. Fourth, while the questionnaire was adapted and assessed for test–retest reliability, it was not fully validated psychometrically. For example, construct validity—such as through exploratory or confirmatory factor analysis—was not evaluated. The test–retest reliability was examined in a subsample of 56 participants, which is adequate for assessing temporal stability but insufficient for full structural validation. Larger samples are needed in future studies to enable comprehensive psychometric testing. Fifth, although the barrier assessment tool was broad in scope, it may not have captured all gender-sensitive or culturally specific barriers. Future research could benefit from mixed-methods approaches—combining surveys with interviews or focus groups—to explore personal narratives and contextual factors, particularly among women. It would also be valuable to assess the effectiveness of targeted interventions aimed at reducing specific barriers, such as expanding women-only facilities, increasing the visibility of female role models, and enhancing peer support networks. Likewise, exploring the influence of institutional policies, media representation, and community initiatives could help identify scalable strategies that foster more equitable participation in PA. Moreover, longitudinal studies should investigate how major life transitions—such as entering the workforce, marriage, or parenthood—affect PA patterns and perceptions of gender-related barriers. It is also important to acknowledge that, while this sample is representative of university students in Riyadh, the findings may not be generalizable to all Saudi youth. Cultural, socioeconomic, and environmental differences across the country should be considered. Broader population-based studies across diverse regions are needed to validate and extend these findings. Finally, the regression models showed modest predictive power, indicating that the perceived barriers measured explain only a limited part of physical activity behavior. Although the questionnaire covered diverse domains—practical, personal, social, cultural, environmental, health-related, social support, and time constraints—other factors like deeper psychological constructs, family dynamics, and broader cultural or environmental influences likely also play important roles. Moreover, psychosocial factors—such as mental well-being or motivation—were not assessed, nor were their potential gender-specific interactions with perceived barriers examined, which limits a comprehensive understanding of these relationships. Future research should include these variables to better understand the complex determinants of physical activity in this population.

## 5 Conclusion

This study highlights that gender differences in perceived barriers to PA among college students in Riyadh are most pronounced across Personal, Social and Cultural, Environmental, and Health-Related domains. Moreover, the frequency of PA engagement is negatively influenced by competing responsibilities, extreme weather conditions, transportation difficulties, and physical fatigue. In contrast, the type of PA—particularly organized and competitive forms—is shaped by barriers such as environmental pollution, the absence of same-gender role models, time management difficulties, limited group support, and insufficient knowledge or skills. While several of these gender differences were statistically significant, the effect sizes were generally small, suggesting that although gendered experiences are relevant, these differences are modest and should be interpreted with caution—especially when guiding broader interventions. Likewise, the modest predictive power of the regression models indicates that additional unmeasured factors likely contribute to PA behaviors in this population.

Based on these findings, promoting equitable PA participation requires the development of female-friendly environments that are perceived as safe and free from harassment. Addressing safety concerns and experiences of discomfort—particularly in public or mixed-gender settings—is essential for building women's confidence in engaging in PA. Strengthening peer support systems, addressing body image concerns, and enhancing the visibility of female role models are also critical components. Additionally, expanding access to appropriate equipment and green spaces may further support motivation and confidence. Practical barriers related to time constraints, fatigue, and lack of skills should be tackled through comprehensive strategies that include educational initiatives and support for time and energy management. Targeting these key barriers may foster more inclusive and sustained participation, contributing to reducing gender disparities in both the quality and type of physical activity practiced.

## Data Availability

The raw data supporting the conclusions of this article will be made available by the authors, without undue reservation.
