# Supplementary material for: Identifying barriers to physical activity participation and engagement among college students in Riyadh (Saudi Arabia): gender differences in perceived barriers
Source: Front Sports Act Living. 2025 Sep 11;7:1657854. doi: 10.3389/fspor.2025.1657854 (PMC12460344; doi:10.3389/fspor.2025.1657854)
Supplement: Supplementary file 1 [file Table1.docx]

**QUESTIONNAIRE**

**Practical Barriers**

1. **Economic access:**
   - **Question:** The costs associated with physical activity are a barrier to my participation.
   - **Responses:**
     - 1: Economic access is a major problem.
     - 5: Economic access is not a problem at all.
2. **Domestic demands:**
   1. **Question:** Household responsibilities and family care prevent me from having time for physical activity.
   2. **Responses:**
      1. 1: Domestic demands completely prevent me from physical activity.
      2. 5: Domestic demands do not affect my physical activity at all.
3. **Transportation:**
   1. **Question:** Lack of safe or accessible transportation limits my ability to attend physical activity facilities.
   2. **Responses:**
      1. 1: Lack of transportation significantly limits my participation.
      2. 5: Transportation is not an issue for me.
4. **Availability of facilities:**
   1. **Question:** I do not have enough physical activity facilities in my community to meet my needs.
   2. **Responses:**
      1. 1: There are no suitable physical activity facilities in my area.
      2. 5: There are plenty of physical activity facilities that meet my needs.
5. **Lack of programs or clubs:**
   1. **Question:** The absence of programs or clubs that encourage my participation in physical activity is a barrier.
   2. **Responses:**
      1. 1: The lack of programs/clubs is a major barrier.
      2. 5: There are many programs/clubs that encourage my participation.

**Personal Barriers**

**2.Body image:**

- - **Question:** I worry about how others perceive me when I engage in physical activity.
  - **Responses:**
    - 1: I am very worried about how others perceive me.
    - 5: I do not worry about how others perceive me at all.

1. **Self-confidence:**
   1. **Question:** I lack confidence to participate in physical activity due to how I feel wearing physical activity clothes.
   2. **Responses:**
      1. 1: My lack of confidence stops me from participating.
      2. 5: I feel confident participating in physical activity.

**16.Knowledge and skills in physical activities:**

- 1. **Question:** My lack of skills or knowledge of physical activities prevents me from participating.
  2. **Responses:**
     1. 1: My lack of knowledge is a big barrier.
     2. 5: I feel I have enough knowledge to participate.

1. **Proper equipment:**
   1. **Question:** The lack of proper equipment limits my ability to engage in physical activity.
   2. **Responses:**
      1. 1: Not having the right equipment is a significant limitation.
      2. 5: I have all the equipment I need for physical activity.

**30.Pressure about social image and masculinization:**

- 1. **Question:** I avoid physical activity due to concerns that it may lead to physical changes (e.g., muscle development) that I do not desire.
  2. **Responses:**
     1. 1: I strongly avoid physical activity due to concerns about appearance changes.
     2. 5: I have no concerns about appearance changes from physical activity.

**Social and Cultural Barriers**

**3.Aggressiveness:**

- - **Question:** Excessive aggression in physical activities discourages me from participating.
  - **Responses:**
    - 1: Aggressiveness in physical activities strongly discourages me.
    - 5: Aggressiveness in physical activities does not discourage me.

1. **Competitiveness:**
   1. **Question:** Excessive competitiveness in physical activities demotivates me from participating.
   2. **Responses:**
      1. 1: Excessive competition is a significant disincentive for me.
      2. 5: Competitiveness in physical activities does not discourage me at all.

**17.** **Lack of same-gender role models:**

- 1. **Question:** The lack of role models of my gender in physical activities limits my motivation to participate.
  2. **Responses:**
     1. 1: The lack of role models significantly limits my motivation.
     2. 5: I am not affected by the lack of role models.

**24.** **Limited media representation by gender:**

- 1. **Question:** The representation of individuals and athletes of my gender in the media is insufficient to motivate my participation in physical activities.
  2. **Responses:**
     1. 1: Poor media representation strongly discourages me.
     2. 5: Media representation does not affect my participation.

**31.Harassment in physical activity spaces:**

- 1. **Question:** I worry about the possibility of harassment or inappropriate comments when engaging in physical activities in public spaces.
  2. **Responses:**
     1. 1: I am very concerned about the risk of harassment.
     2. 5: I am not concerned about harassment.

**Environmental Barriers**

**4.Access to green spaces:**

- - **Question:** I do not have access to green spaces (parks, trails) where I can engage in physical activity regularly.
  - **Responses:**
    - 1: I do not have access to green spaces for physical activity.
    - 5: I have ample access to green spaces.

**11.Safety:**

- - **Question:** Concerns about personal safety (e.g., crime, robberies, lack of surveillance in the area) demotivate me from engaging in physical activity in public spaces.
  - **Responses:**
    - 1: Personal safety concerns strongly prevent me from engaging in physical activity outdoors.
    - 5: I have no personal safety concerns when engaging in physical activity.

**18.Weather:**

- - **Question:** The climate in my area limits my ability to engage in physical activity outdoors.
  - **Responses:**
    - 1: The weather greatly limits my ability to engage in physical activity.
    - 5: The weather does not limit me at all.

**25.Environmental pollution:**

- - **Question:** Air quality or pollution in my area limits my ability to engage in physical activity outdoors.
  - **Responses:**
    - 1: Pollution in my area strongly limits my physical activity.
    - 5: Pollution is not an issue for me.

**32.Facility Lighting & Safety Features:**

- - **Question:** I am concerned about the adequacy of lighting and other safety features (e.g., emergency exits, clear pathways) in the physical activity facilities I use.
  - **Responses:**
    - 1: I am very concerned about the lighting and safety features in facilities.
    - 5: I have no concerns about lighting or safety features in the facilities I use.

**Health-Related Barriers**

**5.Health conditions:**

- - **Question:** I have a health condition that limits my ability to engage in physical activity.
  - **Responses:**
    - 1: My health condition completely limits my physical activity.
    - 5: My health condition does not limit me at all.

**12.Fear of injury:**

- - **Question:** Fear of getting injured demotivates me from participating in physical activity.
  - **Responses:**
    - 1: Fear of injury strongly demotivates me from participating.
    - 5: I have no fear of injury.

**19.Fatigue:**

- - **Question:** Frequent fatigue prevents me from engaging in physical activity regularly.
  - **Responses:**
    - 1: I am frequently too tired to engage in physical activity.
    - 5: Fatigue does not affect my physical activity routine.

**26.Physical pain:**

- - **Question:** Physical pain limits my participation in physical activities.
  - **Responses:**
    - 1: Pain completely prevents me from participating.
    - 5: I have no physical pain that affects my participation.

**33.Recovery from injuries or previous conditions:**

- - **Question:** I have had difficulty resuming physical activity after an injury or health issue.
  - **Responses:**
    - 1: Recovery from an injury or condition makes it very difficult to resume physical activity.
    - 5: Recovery does not affect my ability to resume physical activity.

**Social Support**

**6. Lack of family support:**

- - **Question:** I do not receive support from my family for physical activity.
  - **Responses:**
    - 1: I receive no support from my family for physical activity.
    - 5: My family fully supports my participation in physical activity.

**13. Lack of friend support:**

- - **Question:** I do not have friends who motivate me to participate in physical activities or who accompany me.
  - **Responses:**
    - 1: My friends do not motivate or accompany me in physical activities.
    - 5: My friends strongly motivate and accompany me.

**20. Lack of physical activity group support:**

- - **Question:** I do not have a physical activity group or team to support my routine.
  - **Responses:**
    - 1: I have no physical activity group or team to support me.
    - 5: I have a strong physical activity group or team.

1. **Lack of support network:**
   - **Question:** I do not have a social support network (university or work colleagues) that encourages me to participate in physical activities.
   - **Responses:**
     - 1: I do not have a support network.
     - 5: I have a strong support network.

**34.Lack of coach/teacher support:**

- - **Question:** I do not have coaches or teachers who support me.
  - **Responses:**
    - 1: I do not have coaches or teachers supporting me.
    - 5: I have supportive coaches or teachers.

**Time Constraints**

1. **Lack of time (Family responsibilities):**
   - **Question:** Family responsibilities prevent me from finding time for physical activity.
   - **Responses:**
     - 1: Family responsibilities completely prevent me from physical activity.
     - 5: Family responsibilities do not affect my ability to engage in physical activity.

**14. Lack of time (Academic demands):**

- - **Question:** Academic demands prevent me from finding time for physical activity.
  - **Responses:**
    - 1: Academic demands completely prevent me from physical activity.
    - 5: Academic demands do not affect my ability to engage in physical activity.

**21. Lack of time (Work obligations):**

- **Question:** My work responsibilities leave me little time for physical activity.
- **Responses:**
  - 1: Work obligations greatly reduce the time I can engage in physical activity.
  - 5: Work obligations do not limit my physical activity time.

1. **Other priorities:**

- **Question:** I have other responsibilities, besides family, studies, and work, that are more prioritized than engaging in physical activity.
- **Responses:**
  - 1: Other responsibilities are much more important than engaging in physical activity.
  - 5: Engaging in physical activity is my top priority.

1. **Time optimization:**

- **Question:** I could manage my time better to include physical activity, but my daily obligations make it difficult.
- **Responses:**
  - 1: It is very difficult to manage my time for physical activity.
  - 5: I manage my time well and can always include physical activity.

**Physical Activity Engagement**

1. **Weekly frequency of physical activity:**

- **Question:** How often do you engage in physical activity each week?
- **Responses:**
  - 1: Not at all (0 times a week)
  - 2: Once a week
  - 3: Twice a week
  - 4: Three times a week
  - 5: Four times a week
  - 6: Five times a week
  - 7: Six times a week
  - 8: Seven days a week

**37. Type of physical activity practice:**

- **Question:** How would you describe your level of participation in physical activity?
- **Responses:**
  - 1: I do not practice at all
  - 2: I practice occasionally (recreationally)
  - 3: I engage in physical activity regularly (informal)
  - 4: I participate consistently in organized physical activities
  - 5: I practice at a federated (competitive) level

**Personal Data:**

- - 1. **Age** (in years): ………
    2. **Gender**:

Female

Male
